# Important Elements and Features of Neighborhood Landscape for Aging in Place: A Study in Hong Kong

**DOI:** 10.3389/fpubh.2020.00316

**Published:** 2020-08-17

**Authors:** Shu-Lin Shi

**Affiliations:** Department of Landscape Architecture, School of Architecture, Tsinghua University, Beijing, China

**Keywords:** neighborhood, landscape elements and features, perceived importance, older adults, aging in place

## Abstract

With rapid growth in the aging population around the world, the promotion of aging in place has become more significant in recent years. Many neighborhood landscape elements and features have been revealed by accumulating research findings to be critical to aging in place. However, they are usually studied separately or in small groups. Little has been done to examine the relative importance of these elements and features when brought together, from the older adult's point of view. In this context, the current study investigated the perceived importance for older adults of 22 selected neighborhood landscape elements and features. A questionnaire survey was conducted in 17 public rental housing estates in Hong Kong with proportions of older residents (aged 65 or above) between 20 and 40%. According to the 426 collected samples, older adults considered as highly important landscape elements and features that contribute to comfort and help them avoid hazards, such as good ventilation, protection from severe sunshine/rain, body support, and good hygiene, while elements were thought to potentially bring hazards while not being necessities for older adults' outdoor experience were considered least important, including portable chairs, outdoor tables, plants that can be touched, closeness to children's playgrounds, small spaces for solitude, water features, and fitness equipment. After integrally interpreting the findings regarding perceived importance with other collected data, some landscape design suggestions are generated to supplement existing guidelines and recommendations concerning older adults' well-being and quality of life. These findings can inspire future research and landscape design that prioritize promoting aging in place.

## Introduction

The world's population is aging rapidly. In 2019, about 9% of the global population was aged 65 or above. This proportion is estimated to reach 12% by 2030 and 16% by 2050. Furthermore, projections indicate that the population of aged people will be twice as many as that of children aged 0–4 and will exceed youths aged 5–14 and 15–24, respectively, by 2050 (1). Although an aging population places increasing demands on caring for older adults, it does not make sense to institutionalize them all. In fact, most older adults would prefer not to leave communities that they are familiar with (2). Considering the decaying health condition of most older adults, outdoor space near their residences would play an important role in promoting aging in place, i.e., to support them in living independently in their neighborhoods and homes for as long as possible.

Since the 2000s, there has been a sharp increase in research in this field (3). On the one hand, functional and cognitive impairment, chronic diseases, a diminishing social network, and a low level of physical activities have been identified as hindered aging in place (4–7). On the other hand, neighborhood outdoor environments have been revealed as better for older adults' well-being through helping retain their preferred lifestyles, social connections, and sense of control, together with better clinical outcomes compared with their institutionalized counterparts (8). If an environment is enjoyable, and hence induces enjoyable activities inside it, it would contribute to users' quality of life (9, 10). In such relatively small-scale outdoor environments, landscape elements, and features can be closely experienced and thus be critical for promoting aging in place.

## Outdoor Landscapes for Older Adults

According to mounting research findings, outdoor spaces, especially those with natural elements, have been broadly proven to be contributive to older adults' physical, mental, and social well-being and to further enhance their quality of life (11–15). Actually, once they have stepped into nature, older adults may immediately feel relieved, away from the indoor sources of depression (16). In most cases, the neighborhood outdoor landscape contains natural elements such as plants and water and likely attracts small animals. These are good sources of various sensory stimulations. According to Cox et al. (17), less sadness is experienced when being in a garden or a place with sensory stimulations than when indoors. Even for individuals with dementia, their psychological symptoms decrease after accessing a natural setting (18). If the landscape is properly designed and equipped with facilities, it can encourage physical activities that can sustain and even improve the physical and mental health of older adults (19). In many cases, simply walkable green spaces near residences can positively influence the longevity of older adults in urban areas (20). In addition, inter-personal interactions and activities in the outdoor spaces can provide social support to older adults, thus fostering a sense of belonging or community that can be good for their well-being as well (21).

Although simply viewing a landscape with natural elements is already contributive to human beings' well-being (22–24), there will be much greater well-being benefits and a richer experience when a person is physically in a space with landscape design. Therefore, accessibility and safety should be ensured first to support older adults with declining health conditions (25, 26). Accessibility generally refers to the availability of spaces and certain facilities, connectivity with destinations, and barrier-free design solutions (27). Safety mainly concerns crimes and accidents while using outdoor spaces (28, 29).

Aside from such fundamental factors, landscape design elements have also been discussed in responding to older adults' specific needs. For instance, with reduced strength and stamina, older adults may not be able to walk as far or as fast as younger ones. Correspondingly, they need more resting facilities, shelters, and shade where they can take rests and be protected from unfavorable weather conditions (10). They may also have difficulties in keeping good balance and thus need handrails or other facilities as support (30). Furthermore, many older adults have deteriorated eyesight or visual impairments. This, together with reduced balance, make it easy for them to fall and get hurt due to unleveled pavements, illusions of level changes due to shadows or different colors of paving materials, or glare from paving materials (30). In addition, life after retirement usually makes older adults feel bored, and they need some interests in life or to engage in social activities (31). In supporting these needs, ornamental plants can not only provide sensory stimulations and enjoyments but can also trigger certain interactions between people, thus enhancing social networking (10, 32). People moving around and children playing in a neighborhood also effectively add liveliness to the spaces. Older adults commonly like to watch these people, and may incidentally meet friends, which also contributes to their quality of life (33). Besides, if outdoor space design could provide older adults with more of a sense of control and choice, they would get more satisfaction. For instance, portable chairs that allow people to sit in the positions and orientations they like, tables that provide support for food and drinks or reading, and different paths to take could be included (30).

Based on a growing body of evidence, design guidelines on outdoor landscapes for older adults have emerged. Most guidelines cover the above-mentioned design aspects and provide practical recommendations (30, 34, 35). However, with the constraints of site conditions and available resources for each project, it can be very challenging to meet all design requirements or recommendations in practice, except for some fundamental requirements like barrier-free design, leveled pavements, and sufficient lighting. Usually, it is also hard to judge which landscape elements and features are more important, as most of them are studied separately by different researchers and with different research methodologies. These may hinder the utilization and well-being benefits of landscape (36, 37). The above review implies that older adults' concerns related to community landscape design should be studied comprehensively. For instance, when different landscape elements and features are put together, which of them are more important for older adults? Therefore, this study was conducted to investigate the perceived relative importance of landscape elements and features in the eyes of older adults, focusing on neighborhood outdoor landscapes, with the hope of supporting landscape research and design that aims to promote aging in place.

## Methodology

### Hong Kong Situation

The study was conducted in Hong Kong, a city facing serious aging problems, like many other cities in the world. According to the Census and Statistics Department of the HKSAR government, the proportion of people aged 65 and above reached 17.0% of the entire population in mid-2018 (38), and it is expected to reach 31.1% (2.37 million in total) by 2036 and 36.6% (2.59 million) in 2066 (39). As revealed by a government survey, older adults in Hong Kong would like to age at home and live in a familiar community until they need residential care services: 96.4% of 1,130 elderly participants did not intend to move into a local residential elderly care facility (40). This is supported by findings of a more recent survey in Hong Kong (41).

With the dense and compact development mode in Hong Kong, outdoor spaces within residential estates are critical for aging in place and the well-being of older adults, as most of them are traffic-free. Among different types of residential developments in Hong Kong, public rental housing (PRH) estates developed by the Hong Kong Housing Authority (HA) and the Hong Kong Housing Society (HS) always provide such spaces at a relatively sufficient proportion of the total site area. Meanwhile, the HA and HS are also major providers of rental housing for senior citizens in Hong Kong. According to the 2016 Population By-census, among the 2,100,126 total population living in PRH, 392,575 (18.7%) were older adults aged 65 or above, comprising 36.7% of all elderly people in domestic households in Hong Kong (42). The HA and HS's role in supporting the aging population will become more critical in future, and so will outdoor landscape design in PRH estates.

### Estate Selection

**Figure 1:**
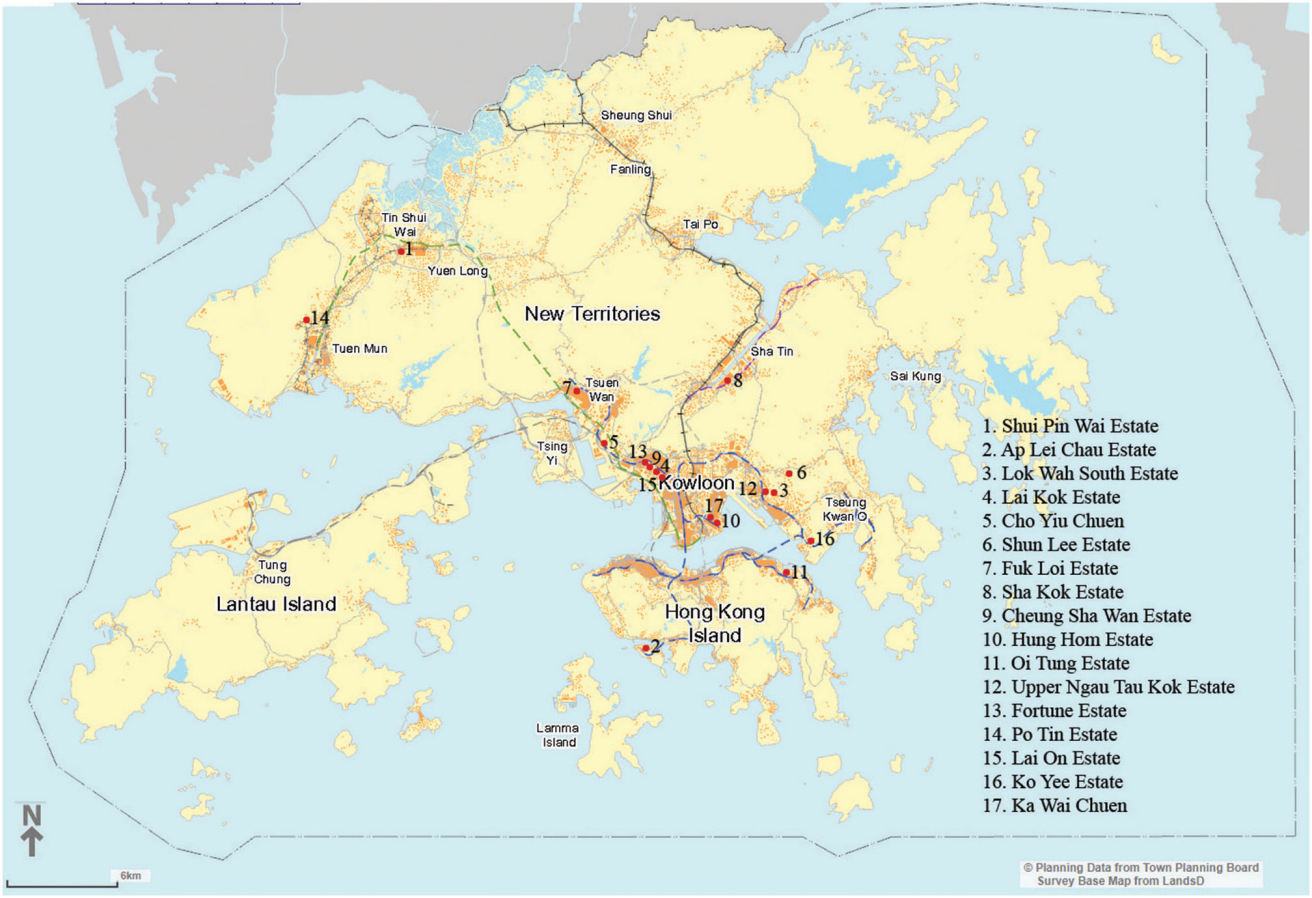
Distribution of selected PRH estates (Source of base map: https://www1.ozp.tpb.gov.hk/gos/default.aspx).

**Figure 2:**
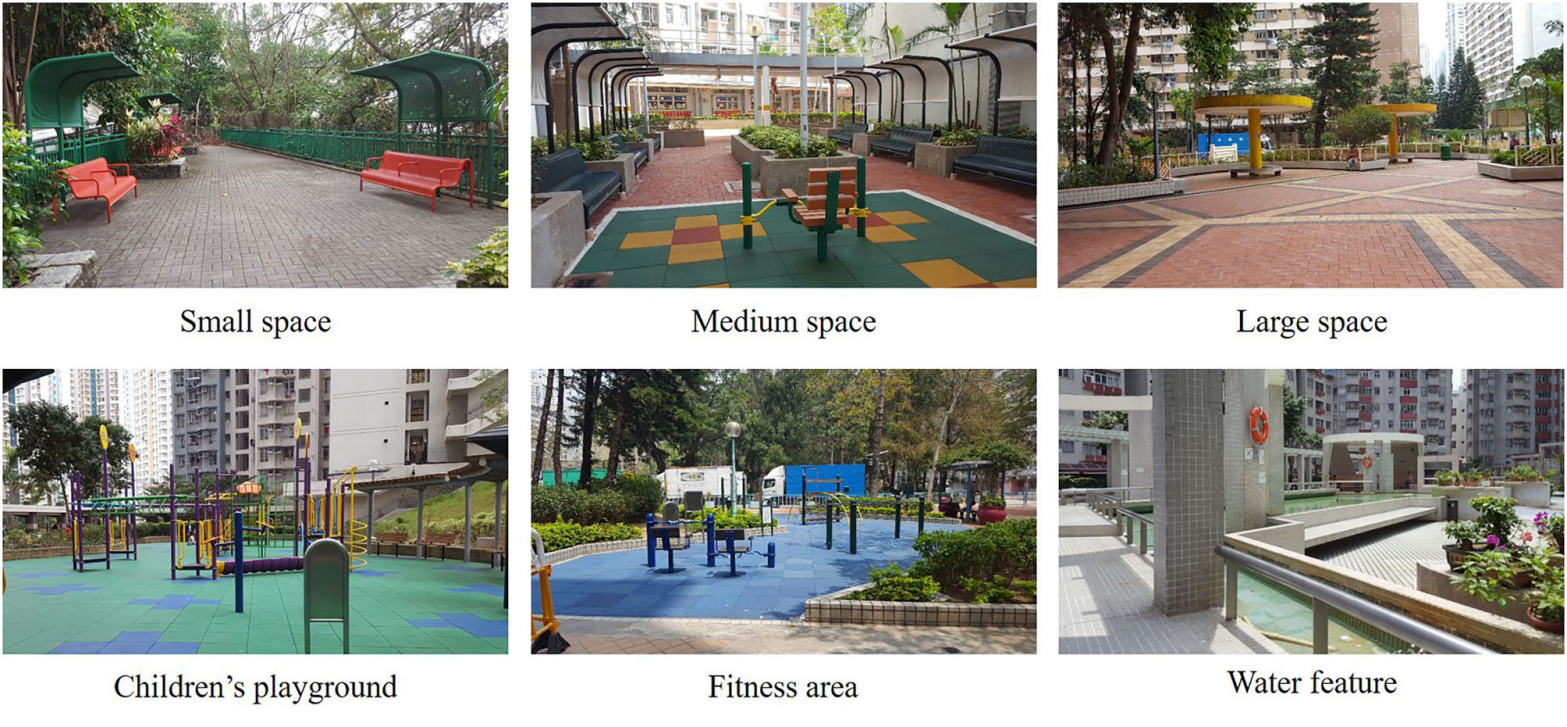
Typical landscape elements and features in selected PRH estates (photoed by author).

Among the 199 PRH estates in Hong Kong (190 developed by the HA and 9 by the HS), most open spaces were developed in similar ways, except for accommodating fewer water features in recent years and some style evolutions on plant species selection (43). In this study, 17 PRH estates were selected, mainly based on the proportion of aged residents and forward-looking considerations: eight with 30–40% aged people among residents, representing an overall population scenario in the next half-century or even a longer period, and the remaining nine with 20–30% aged people among residents to represent a scenario of the near future (Table 1). The distribution of these estates is shown in Figure 1. Examples of typical landscape elements and features in these estates are shown in Figure 2.

**Table 1:** Basic information of 17 selected PRH estates.

**No**.	**Authorized party**	**Name of estate**	**Year of intake (establishment)**	Population aged 65 and above^*^	Total population^*^	**% of aged residents**
1	HA	Shui Pin Wai Estate	1981	2,691	6,725	40.0
2	HA	Ap Lei Chau Estate	1980	5,272	14,504	36.4
3	HA	Lok Wah South Estate	1982	4,452	12,843	34.7
4	HA	Lai Kok Estate	1981	2,224	6,488	34.3
5	HS	Cho Yiu Chuen	1976/78/79/81	2,424	7,159	33.9
6	HA	Shun Lee Estate	1978	4,110	12,363	33.2
7	HA	Fuk Loi Estate	1963	2,260	6,999	32.3
8	HA	Sha Kok Estate	1980	4,384	14,522	30.2
9	HA	Cheung Sha Wan Estate	2013	800	3,344	23.9
10	HA	Hung Hom Estate	1999/2011	1,553	6,623	23.5
11	HA	Oi Tung Estate	2001	2,254	8,028	28.1
12	HA	Upper Ngau Tau Kok Estate	2002/09	4,151	15,004	27.7
13	HA	Fortune Estate	2000	1,115	4,489	24.8
14	HA	Po Tin Estate	2000	2,174	10,782	20.2
15	HA	Lai On Estate	1993	715	2,957	24.2
16	HA	Ko Yee Estate	1994	761	3,326	22.9
17	HS	Ka Wai Chuen	1984/87/90/93	1,511	6,928	21.8

* *According to population census 2016 (44)*.

### Questionnaire Design

A questionnaire was designed to collect data mainly about perceived importance for a set of landscape elements and features in neighborhood outdoor spaces. The landscape elements and features were extracted from various design guidelines and published studies concerning outdoor landscape elements and features for aged people, while those already commonly agreed as fundamental and that have been incorporated in PRH estates, such as barrier-free accessibility and a non-slippery ground surface, were excluded. Each of the 22 final listed items' association with the well-being and quality of life of aged people has been identified or proven by empirical studies. These landscape elements and features were categorized into Convenience, Comfort, Sense of safety, Sense of control, Stimulation, and Social support to facilitate discussion (Table 2).

**Table 2:** Selected landscape elements and features.

**Category**	**Sub-category**	**Landscape elements/features in questionnaire**	**Studies on health influence & design guidelines of landscape element/feature**
Convenience	Supportive distance	Close to major pedestrian route	(45)
		Close to shops (convenient purchase of food and drinks)	(46) (47) (48) (49) (50)
		Close to public toilet	(10, 12)
	Supporting elements	Body support (bench, planter edge, railing, etc.)	(51) (30)
		Outdoor table (independent or attached to a bench)	(30)
Comfort	Weather-related solutions	Avoidance of severe sunshine/rain	(10) (52)
		Good ventilation	(49)
	Hygiene	Good hygiene	(49)
Sense of safety	–	Can see what is happening from outside(to decide whether to enter or not, timely emergency treatment, etc.)	(37)
		Can see what is happening nearby	(37)
		Quiet environment	(49)
Sense of control	Privacy	Small space for solitude	(30) (53)
	Have choices	Multiple entrances/exits for a space	(30)
		Portable chair	(30) (53)
Stimulation	Sensory stimulation	Open view (can see distant plants, buildings, mountains, etc.)	(30) (53)
		Water feature (pool or water fountain, etc.)	(30) (53)
		Ornamental plants	(27) (10) (54) (32)
		Plants that can be touched	(30) (55)
		Visible dynamic elements (e.g., activities of other people, people/vehicles passing by, small animals, dynamic water, water fountain, plants moving in the wind, etc.)	(30)
	Exercise stimulation	Fitness equipment	(19) (56) (49)
Social support	–	Large space for gathering	(57) (49)
		Close to children's playground	(10)

Specifically, Convenience covers two sub-categories, namely, supportive distance, and supporting elements. Supportive distance mainly concerns the distance between major destinations and facilities that older adults would need to visit, e.g., shops and public toilets; together with allocation of neighborhood outdoor spaces, i.e., the distance between spaces and major pedestrian routes (45, 46, 49, 50). Supporting elements refers to those that have been shown by some studies to help older adults keep their body balance and avoid falling (51). However, in Hong Kong, older adults tend to equip themselves with canes, walkers, and wheelchairs to avoid potential falls. Hence, supporting elements in this study refers to body support, like benches, planter edges, and railings, or elements like outdoor tables that help relieve users' burden (30, 51).

Comfort covers aspects of weather-related solutions and hygiene. The sub-tropical climate in Hong Kong makes it hot and humid, with a lot of showers, during long summers. Therefore, elements that can protect people from severe sunshine/rain and spaces with good ventilation (air flow) are critical to enable people to stay outside (10, 49, 52). In addition, hygiene would also affect people's comfort through visual and osphretic aspects (49). Poor hygiene conditions may also spread germs and affect the health of vulnerable older adults. This is also a concern of older PRH residents (41).

Regarding Sense of safety, it is generally quite safe in PRH estates, as security guards patrol frequently and CCTV covers all public areas. Therefore, safety in this study is mainly about whether older adults can receive instant help in case of accident and whether they are forewarned of potential hazards. Under such circumstances, the in-outward visual connections of a space could be critical (37). Besides, quiet environments can lower the alert level and make people feel safe and relaxed (49). Although it is arguable that people may feel upset in a completely quiet environment, such a case seldom exists in outdoor spaces in developed areas in Hong Kong due to its high population density.

Research has found that older adults with a higher sense of control usually enjoy better health (58). Therefore, it would be good if outdoor landscapes could provide a certain extent of privacy by creating small spaces where one or two people could stay alone and away from disturbances (30, 53). Besides, providing different route choices to a space can also enhance the sense of control (30). In addition, portable chairs are also considered contributive to sense of control, as discussed above (30, 53).

The category of Stimulation covers sensory and exercise stimulations in this study. Sensory stimulations include landscape elements and features like open views, water features, ornamental plants, plants that can be touched, and visible dynamic elements (10, 27, 30, 32, 53). Among these, touching plants has been proven to be soothing and therapeutic (55). However, to prevent mosquito problems, most landscape property managements apply pesticides on plants and warn people about this with signage boards in Hong Kong. Therefore, Hong Kong people tend not to touch plants in reality. Nonetheless, behavior may not reflect attitudes or willingness. Therefore, this item is still included to examine older adults' attitudes. Additionally, fitness equipment is included under exercise stimulation as facilitators of physical activities for older adults (59–62).

Social support mainly refers to meeting with people and friends in the neighborhood, such as gathering with friends in relatively large outdoor spaces in the estates, or watching children play, which add liveliness in older adults' lives (10, 57). It is critical for older adults to keep connections with others in society (57). Among various connections, intergenerational programs have become popular among caregiving organizations for older adults (63). Although positive intergenerational relationships are mainly observed in elderly caring facilities, it is suspected to be valid in neighborhoods with mixed generations as well.

For each of the 22 landscape elements and features, participants need to assign a mark on a scale from 0 to 10, where a higher mark represents higher perceived importance. To assist in analyzing the possible reasons for perceived importance, the time and days that older people would use neighborhood outdoor spaces and their outdoor activities within their estates were also investigated in the questionnaire. Activities were first extracted from the author's previous studies on older adults in Hong Kong (36, 37) and were supplemented by pilot studies in the selected PRH estates. These activities were shown in a multiple-choice question in the questionnaire. Participants could tick or supplement to indicate all of their activities in neighborhood outdoor spaces. In addition, demographic information such as age, gender, self-evaluated health, physical impairment, household composition, and period living in the estate was also collected.

### Data Collection and Processing

**Figure 3:**
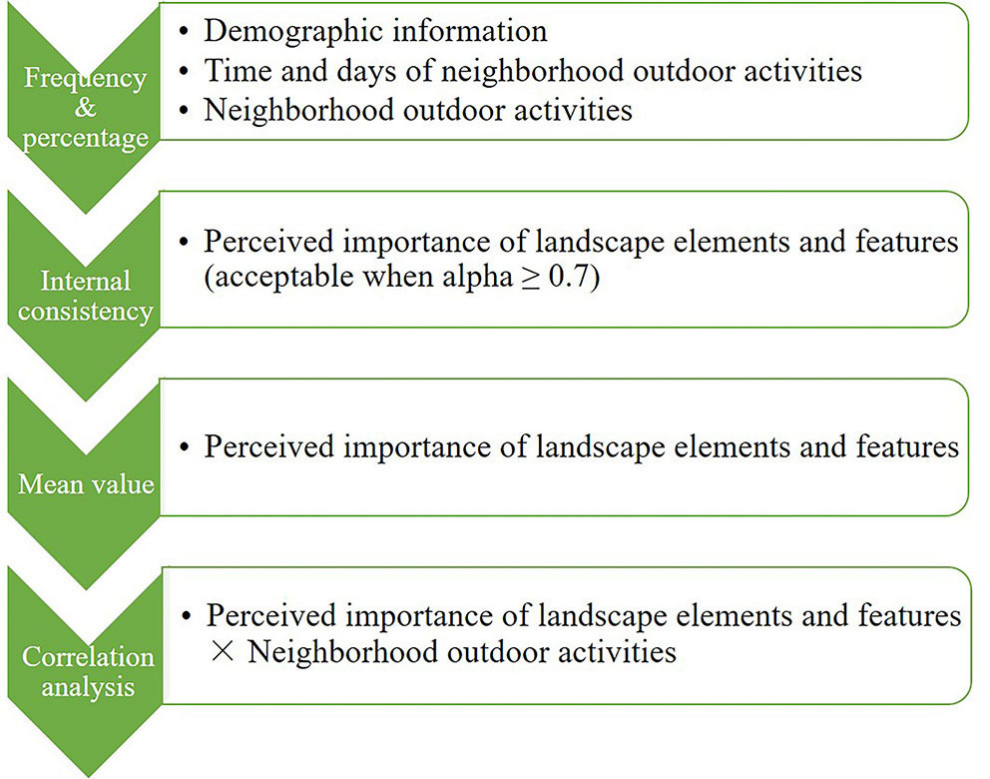
General analysis process.

The survey was conducted during June and July 2019 in the selected 17 PRH estates. During the survey, people seemingly aged 65 or above were approached randomly and were filtered out if they were not residents in the current estate or aged under 65. During the survey, participants were encouraged to share reasons for their answers and insights into their neighborhood outdoor spaces. Participants were allowed to quit during the survey as they liked. The planned sample size in each estate follows the proportion of the older-resident population in that estate to the sum of those in all 17 estates, based on a total targeted sample size of 420 [considering potential loss in sorting, the targeted sample size was set as 1.1 times the original 381, which was calculated with 5% confidence interval, 95% confidence level, and a target population of 42,851 (64), and rounded up to nearest 10]. Collected data were processed with IBM SPSS 22. The general analysis process is shown in Figure 3 and will be elaborated on below.

## Results

A total of 426 valid samples were collected. Among these, 241 were female (56.6%) and 185 were male (43.4%). This is generally consistent with the overall demographic situation among older adults in Hong Kong by mid-2018 (65). Of the participants, 41.5% claimed that they did not have any chronic diseases; 12.9% thought their health condition was very good, 46.9% good, 33.1% generally ok, and 7% poor or very poor. The most mentioned chronic diseases were cardiovascular and cerebrovascular (39.9%), followed by orthopedics diseases (36.1%) and diabetes (9.6%). In terms of physical impairment, 58.2% of participants claimed that they did not suffer from any such impairment. Among various impairments, impaired mobility was most common (31%). Regarding household composition, 34.5% of participants lived alone, while the rest lived with other people like children, spouses, parents, and domestic helpers.

By the time of this survey, 99.3% of participants had lived in their estates for more than 1 year and 84% for more than 10 years. Most participants used open spaces in their estates frequently: 63.6% used such spaces more than once every day, 27.9% once every day, and 8.4% once to twice a week or less. They visited these outdoor spaces on any day throughout a week, except a bit less on public holidays (88.5%). Considering a single day, around 70% participants visited the outdoor spaces during 9 a.m.−12 noon and 3–5 p.m., around 40% came out in early mornings before 9 a.m., ~20% stayed outside during noon hours, while <20% stayed outside after 6 p.m. These responses indicate that most participants visited outdoor spaces in their estates frequently and were familiar with these spaces and the facilities within them and thus that their replies to our survey could be considered reliable.

**Figure 4:**
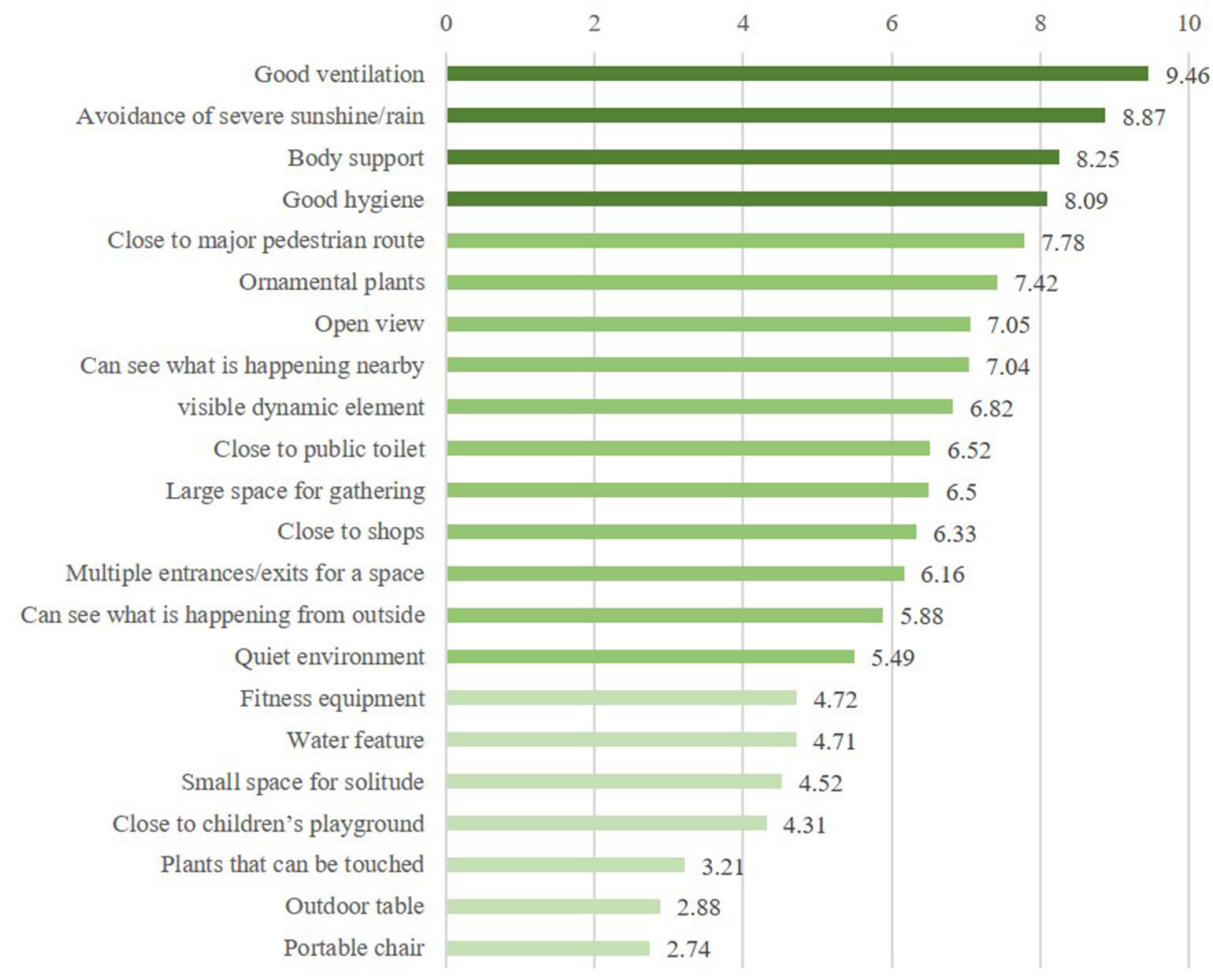
Perceived importance of each landscape element/feature (ranked).

The internal consistency of the 22 landscape elements and features was examined with a reliability analysis in SPSS. The result for Cronbach's alpha is 0.713, which indicates an acceptable internal consistency among the 22 items. The perceived importance of the 22 landscape elements and features is represented by the mean values of the item scores received (Figure 4). Generally speaking, landscape elements and features that related to Comfort, i.e., good ventilation, avoidance of severe sunshine/rain, and good hygiene, together with body support under Convenience fall into the high range (>8.0), except that in Comfort, a few items under each of the rest categories fall into the medium (5.0–8.0) and low (<5.0) ranges in terms of perceived importance among participants. Additionally, the frequency and percentage of each type of activity are summarized in Table 3. Those taken part in by 50% or more of the participants are mainly passive activities.

**Table 3:** Older residents' activities in outdoor spaces in PRH estates (popular activities are shown in bold).

	**Passing by**	**Sitting**	**Watching others' activity**	**Chatting**	**Waiting for others**	**Basking**	**Enjoying the cool**	**Reading/writing/drawing/watching mobile**	**Resting/napping**	**Contemplation**	**Eating/drinking**	**Smoking**
Frequency	84	**391**	**239**	**324**	19	62	**312**	59	71	6	40	35
Percent	19.7	**91.8**	**56.1**	**76.1**	4.5	14.6	**73.2**	13.8	16.7	1.4	9.4	8.2
	**Watching plants/animals**	**Listening to animals**	**Leisure walking**	**Stretching**	**Exercising**	**Walking exercise**	**Taking care of grandchild**	**(Watching) chess/card playing**	**Listening to music/opera**	**Singing/playing instrument**	**Drying clothes**	**Other**
Frequency	**209**	166	**215**	150	108	49	6	39	46	3	12	13
Percent	**49.1**	39	**50.5**	35.2	25.4	11.5	1.4	9.2	10.8	0.7	2.8	3.1

## Discussion and Design Implications

### Landscape Elements and Features With High Perceived Importance

Based on literature review, landscape elements and features that related to Convenience were expected to receive higher rankings in perceived importance. Surprisingly, only Body support under this category was scored as having high perceived importance, while all three items under Comfort were scored highly. To explore the possible reasons for this, correlations between items with perceived importance >8.0 and activities conducted by participants were examined. According to Table 4, activities that significantly correlated with items under Comfort were relatively more popular among participants in PRH outdoor spaces (see Table 3). This indicates a high representativeness of these correlations among older adults in their residential outdoor spaces in general.

**Table 4:** Correlation between items with perceived importance >8.0 and activities (significant correlations are shown in bold).

	**Passing by**	**Siting**	**Watching others' activity**	**Chatting**	**Waiting for others**	**Basking**	**Enjoying the cool**	**Reading/writing/drawing/watching mobile**	**Resting/napping**	**Contemplation**	**Eating/drinking**	**Smoking**
Good ventilation	−0.114^*^	0.152^**^	0.190^**^	−0.003	−0.056	0.096^*^	0.302^**^	0.060	0.063	−0.013	0.023	0.020
Avoiding severe sunshine/rain	−0.030	0.163^**^	0.233^**^	0.116^*^	0.059	−0.092	0.268^**^	0.076	0.010	−0.002	0.108^*^	0.057
Body support	−0.063	0.192^**^	0.177^**^	0.087	−0.125^**^	0.035	0.317^**^	0.058	−0.011	−0.004	0.040	−0.009
Good hygiene	−0.005	−0.008	−0.067	0.007	0.002	0.085	0.085	−0.050	0.198^**^	0.038	0.050	−0.015
	**Watching plants/animals**	**Listening to animals**	**Leisure walk**	**Stretching**	**Exercising**	**Walking exercise**	**Taking care of grandchild**	**(Watch) chess/card play**	**Listening to music/opera**	**Singing/playing instrument**	**Drying clothes**	**Other**
Good ventilation	0.208^**^	0.169^**^	0.252^**^	0.153^**^	0.040	−0.011	0.037	0.013	0.074	−0.080	0.017	0.057
Avoiding severe sunshine/rain	0.151^**^	0.158^**^	0.190^**^	0.057	0.045	0.045	−0.022	0.105^*^	0.052	−0.106^*^	−0.052	−0.043
Body support	0.189^**^	0.206^**^	0.207^**^	0.083	0.114^*^	0.042	0.027	−0.011	0.042	−0.040	−0.032	−0.038
Good hygiene	0.136^**^	0.097^*^	0.114^*^	0.041	0.103^*^	0.082	−0.082	−0.025	0.049	−0.096^*^	0.030	−0.106^*^

* *p < 0.05 (2-tailed)*,

** *p < 0.01 (2-tailed)*.

Good ventilation was negatively correlated with Pass by, which implies that if participants find that the ventilation of a space is good, they tend to stay in it. Furthermore, good ventilation promotes various relaxing and passive activities, like enjoying the cool, watching plants/animals, watching others' activity, and listening to animals. Under such circumstances, people may have an experience similar to meditation and be more sensitive to direct sensations (66). During hot days, good ventilation with breezes can effectively strengthen evaporation on the skin, hence improving thermal comfort (67). This could encourage leisure walking and stretching as well as outdoor activities in general among older adults.

Avoidance of severe sunshine/rain in outdoor spaces seems to be more influential on activities with a potentially long duration, like sitting, watching others' activity, chatting, enjoying the cool, watching plants/animals, listening to animals, and leisure walking. It appears critical to activities that rely heavily on vision, such as eating/drinking and playing chess/cards, as older adults can be easily affected by strong light or glare due to deteriorated vision and visual impairment (68). Besides lighting conditions, rain can also affect older adults' use of outdoor spaces. Most of the participants told us that they would stay indoor if it was raining. However, if a shower came when they were already outside, they would be easily caught by the rain, as many of them move slowly and are afraid of falling due to rushing or the wet ground. Therefore, if there are shelters that can protect them from the showers, older adults can enjoy their outdoor activities without worrying about sudden changes in the weather.

High perceived importance of Good hygiene is probably rooted in participants' consciousness that a space with poor hygiene could harm their health. Since maintaining health is critical and even challenging to most older adults, it is understandable that they would not put themselves at such risk. Regarding associations with activities, Good hygiene is positively correlated with rest, watching plants/animals, listening to animals, leisure walking, and exercise. Actually, some participants expressed reservations about birds or other animals, for these animals may spread bird flu or other diseases, and their feces can stain the environment. This could partially explain why older adults emphasize the hygiene of an outdoor space while they enjoy small animals around them.

Another highly scored element is Body support under the category of Convenience. This item covers various seating facilities and alternatives such as planter edges that are suitable for sitting and elements (railings) at waist or back height to support leaning. It is more associated with static activities, especially long-lasting ones, e.g., watching others' activity, enjoying the cool, watching plants/animals, and listening to animals (Table 4). In order to help sustain older adults' self-esteem, it is better to integrate these body support elements into general landscape design instead of making them specifically for older adults.

### Landscape Elements and Features With Low Perceived Importance

Landscape elements and features with an average mark of <5.0 include Fitness equipment, Water feature, and Plants that can be touched under the category of Stimulation, Small spaces for solitude and Portable chairs under Sense of control, Close to children's playground under Social support, and Outdoor tables under Convenience. These outcomes are surprising, as all of these items have been stressed in various design guidelines for older adults (12, 30, 53, 69). Again, associations between these items and activities, together with comments received during the surveys are examined to facilitate interpretation (Table 5).

**Table 5:** Correlation between items with perceived importance <5.0 and activities (significant correlations are shown in bold).

	**Passing by**	**Sitting**	**Watching others' activity**	**Chatting**	**Waiting for others**	**Basking**	**Enjoying the cool**	**Reading/writing/drawing/watching mobile**	**Resting/napping**	**Contemplation**	**Eating/drinking**	**Smoking**
Fitness equipment	0.106^*^	−0.078	−0.133^**^	0.059	−0.040	0.039	−0.059	−0.071	0.050	−0.007	0.011	0.027
Water feature	0.055	0.050	0.036	0.036	−0.055	0.013	0.039	−0.008	0.013	0.085	0.062	0.070
Small space for solitude	0.106^*^	−0.012	−0.070	−0.154^**^	0.089	0.083	−0.117^*^	0.066	0.000	−0.007	−0.067	0.022
Close to children's playground	0.024	−0.083	0.033	0.078	−0.094	0.054	0.040	−0.027	0.025	0.024	0.143^**^	0.030
Plants that can be touched	0.106^*^	−0.078	−0.133^**^	0.059	−0.040	0.039	−0.059	−0.071	0.050	−0.007	0.011	0.027
Outdoor table	0.080	−0.077	−0.043	−0.013	0.153^**^	0.000	−0.114^*^	0.038	0.032	−0.033	−0.081	0.035
Portable chair	0.065	−0.018	−0.103^*^	0.008	−0.029	0.070	−0.066	−0.021	−0.060	−0.070	−0.012	0.095
	**Watching plants/animals**	**Listening to animals**	**Leisure walking**	**Stretching**	**Exerciseing**	**Walking exercise**	**Taking care of grandchild**	**(Watching) chess/card playing**	**Listening to music/opera**	**Singing/playing instrument**	**Drying clothes**	**Other**
Fitness equipment	−0.049	−0.017	−0.127	0.153^**^	0.281^**^	0.021	−0.022	0.049	0.045	−0.016	0.016	0.006
Water feature	0.109^*^	0.138^**^	0.035	0.071	0.170^**^	−0.058	−0.036	0.100^*^	0.009	0.032	0.068	−0.049
Small space for stay alone	−0.055	−0.057	−0.147^**^	−0.094	−0.097^*^	0.054	−0.048	−0.088	0.000	0.012	−0.077	−0.060
Close to children's playground	0.121^*^	0.153^**^	0.053	0.072	0.078	−0.036	0.106^*^	0.011	0.037	−0.008	0.030	0.064
Plants that can be touched	−0.049	−0.017	−0.127^**^	0.153^**^	0.281^**^	0.021	−0.022	0.049	0.045	−0.016	0.016	0.006
Outdoor table	−0.064	−0.039	−0.062	0.002	0.011	0.006	−0.027	0.181^**^	−0.039	0.090	0.046	0.028
Portable chair	−0.117^*^	−0.130^**^	−0.185^**^	0.001	0.034	−0.035	−0.009	0.173^**^	0.020	0.015	−0.061	−0.079

* *p < 0.05 (2-tailed)*,

** *p < 0.01 (2-tailed)*.

Regarding Fitness equipment, some participants commented that it was more suitable for healthy and young people with a higher level of strength. Some also mentioned that many types of fitness equipment were over-sized for them. Older adults who often do stretches and exercise may not have such problems and also use fitness equipment more (Table 5). Nonetheless, demands for properly designed fitness equipment should not be denied. A good approach would be to provide aging-friendly fitness equipment to encourage exercise among older adults and further contribute to their well-being through a more physically active lifestyle (70, 71).

Water features, Close to children's playground, Plants that can be touched, Outdoor tables, and Portable chairs are considered less important by participants mainly due to safety concerns. For Water features, some participants told us that children would play in the water and wet the ground nearby, which would be slippery. Similarly, children may cause other hazards if they play close to older adults. For instance, children would occasionally bump into people when running around. This could injure older adults seriously. One participant told us that she was once knocked down by a child in her estate and suffered a broken bone. Despite these issues, children's contribution to liveliness is highly appreciated in these estates with high proportions of older adults. For Plants that can be touched, relatively active people, like those who take part in exercise or stretching, may be more sensitive to lively elements and appear to consider it somewhat important (Table 5). In reality, few people take action although many said that they would like to touch beautiful or lovely plants if they came across them, mainly due to the application of pesticides discussed above.

Regarding outdoor tables, participants shared that some people partied around them until midnight, made a lot of noises, and dirtied the place; in some other cases, there were quarrels between different people competing to use the tables. In most cases, outdoor tables were dismantled by property management in the end. For portable chairs, even though many participants reflected that seating is inadequate in their estates, they did not like portable chairs. They commonly worried about falling while sitting down or standing up or tripping over while walking if the chairs were not fixed. Seemingly the only case that outdoor tables and portable chairs are in need is for (watching) playing chess/cards (Table 5). This is supported by frequent observations of older adults who play chess/cards on benches or even on planter edges and attract crowds around them.

Considering Small spaces for solitude, four out of five significant correlations between this item and activities are negative (Table 5). This indicates that older adults tend to be aggregation-oriented, even with little interaction. One reason for this would be that many of them live alone (34.5% of the participants in this study). Such an isolated or semi-isolated life could be stressful for many people, especially vulnerable older adults (72, 73). It may also lead to loneliness and depression and affect their quality of life (74). From this perspective, older adults need a sense of been connected with society, which could be largely achieved by spending time with others in neighborhood outdoor spaces. Another possible reason could be that older adults would not feel released in an outdoor space that is similar to or even smaller than their residence in size. Therefore, this study reveals that compared to small spaces with high privacy, older adults prefer spacious ones that can support a certain extent of gathering.

### Landscape Elements and Features With Medium Perceived Importance

The remaining landscape elements and features had medium perceived importance, including Close to major pedestrian route, Close to public toilet, and Close to shops under the Convenience category; Ornamental plants, Open view, and Visible dynamic element under Stimulation; Can see what is happening nearby, Can see what is happening from outside, and Quiet environment under Sense of safety; Large space for gathering under Social support; and Multiple entrances/exits for a space under Sense of control.

Seemingly, items under Convenience and Stimulation are relatively important within this range. They mainly represent supportive distance and the interestingness of views. If considered integrally with Body support, this implies that if sufficient body support elements or facilities were provided, older adults would not mind walking farther. This could be a valuable reference for neighborhood outdoor space design and even for community planning with special concerns about older adults. Views inwards to and outwards from a space could be easily realized simultaneously when creating open views and could be better integrated with locomotional access, i.e., entrances to spaces. Regarding sharing a space with others, older adults in this study showed less concern. This can be explained by their low intention to spend time alone in outdoor spaces as discussed above. However, they seem not to be proactive for Social support either, as many thought themselves too old and weak to engage in organized activities. The only significantly correlated activities with this item are chatting (*r* = 0.333, *p* = 0.000), rest/nap (*r* = 0.125, *p* = 0.010), and exercise (*r* = 0.122, *p* = 0.012). Chatting and exercising are commonly observed to be conducted by different people together, often with interactions. Resting/napping in a crowd or next to other people may contribute to a sense of safety or being connected to the society. It seems that older adults in the studied estates tend to make minimal effort to maintain social connections and avoid loneliness, which has been commonly agreed as a predictor of functional decline (6, 7).

### Design Implications

Our findings regarding the perceived importance of neighborhood landscape elements and features for older adults generate some landscape design suggestions. These could supplement existing guidelines and recommendations concerning aging-friendly landscape design:

- Weather-related solutions such as good ventilation and weather protection should be provided carefully so as to improve the general outdoor experience for older adults. This would be especially important for places with hot seasons and high annual rainfalls.
- Water features and children's playground can add a lot of interest and liveliness to neighborhoods, which could greatly enrich older adults' outdoor experience. However, they may also become hazards to older adults. Therefore, it is better to locate them at a certain distance from major routes and spaces that are heavily used by older adults while providing visual and acoustic connections in between. Level differences, short fences, or hedges between these spaces may help to achieve such safe connections.
- The safety issues of any portable facilities or elements, like portable chairs, should be carefully considered. In order to ensure the safety of older adults while fulfilling the needs of other age groups in neighborhoods, it is suggested to locate such facilities away from major routes and spaces that are used heavily by older adults.
- The boundaries of each single space should be designed to ensure good in-outward visual connections to support timely help when needed and to avoid any corner or spot that may lead to hygiene problems. These approaches can also contribute to security in outdoor spaces, especially those that lack CCTV coverage. It would be good to integrate space boundaries with natural elements so as to enrich interest and strengthen the well-being benefits of outdoor spaces.
- The sizes of facilities should be carefully decided or adjusted for older adults rather than simply applying standard ones. This is especially important for fitness equipment and benches, which could bring a lot of benefits and are in great need.

## Limitations

Neighborhood outdoor spaces are complex systems that involve numerous interactive factors. Although the landscape elements and features investigated in this study are extracted from a comprehensive literature review and are supplemented by initial site observations, there may still be important ones for aging in place yet to be covered. Besides, this study focuses on PRH estates, which leads to a relatively limited diversity in older residents, landscape designs, and property management. If older adults from other socio-economic groups and different types of residential developments such as private ones could be included, the findings would be enriched and more comprehensive for neighborhood landscapes. Furthermore, this study emphasizes the subjective perceptions of participants. The cross-sectional data employed in this study illustrate such perceptions at a certain point in time. However, perceived importance may evolve with the development of the entire society. To further understand the evolution of perceived importance over time, longitudinal data would be necessary. Moreover, the rigor of the study could be strengthened if supported with objective measurements and analyses on the landscape elements and features. When research on this topic goes deeper, different characteristics of older adults could be further discussed to generate more specific and detailed design recommendations. These would be potential directions for future research in this field.

## Conclusion

This study investigated the perceived relative importance of 22 neighborhood landscape elements and features from the perspective of older adults. It reveals that older adults tend to judge the importance of any landscape element or feature upon comprehensive evaluation of the potential benefits and hazards it would bring, especially emphasizing comfort and safety.

Landscape elements or features that contribute to comfort and help avoid hazards, like good ventilation, avoidance of severe sunshine/rain, body support, and good hygiene, are considered highly important by older adults. Sufficient provision and proper design of these are critical for older adults so that they can use outdoor spaces or avoid difficulties in moving around in their neighborhoods or avoid being attacked by germs, which may threaten their well-being and quality of life. Therefore, these are found to be fundamental landscape elements and features for older adults and should be given priority in neighborhood landscape design.

In contrast, any landscape element or feature that may bring hazards while not being a necessity for older adults' outdoor experience is considered least important. This group comprises portable chairs, outdoor tables, plants that can be touched, closeness to a children's playground, small spaces for solitude, water features, and fitness equipment. If safety concerns could be addressed properly, these landscape elements and features would still be appreciated by older adults.

In between the above two clusters are landscape elements and features that are perceived as of medium importance by older adults. They commonly have alternatives and are not considered necessities. Being close to major routes, ornamental plants, open views, visual contacts inward toward and outward from a space, visible dynamic elements, availability of public toilets, a large space for gathering, being close to shops, multiple entrances to a space, and a quiet environment all fall within this group. These elements and features are not that fundamental but would affect the richness and convenience of the outdoor experience of older adults to a certain extent, especially through contact with nature.

Based on our findings of the perceived relative importance of these neighborhood landscape elements and features, some landscape design suggestions were generated to supplement existing guidelines and recommendations concerning older adults' well-being and quality of life. These will be valuable for neighborhood landscape research and for designs that prioritize promoting aging in place effectively.

## Data Availability Statement

All datasets generated for this study are included in the article/supplementary files.

## Ethics Statement

The studies involving human participants were reviewed and approved by the Human Subjects Ethics Sub-committee (HSESC) of THEi, Hong Kong S.A.R. Written informed consent for participation was not required for this study in accordance with the national legislation and the institutional requirements.

## Author Contributions

The author designed the study, participated and monitored data collection, conducted data analyses, data interpretation, and prepared this article.

## Conflict of Interest

The author declares that the research was conducted in the absence of any commercial or financial relationships that could be construed as a potential conflict of interest.
